# Rapid Access to Contrast-Enhanced spectral mammogRaphy in women recalled from breast cancer screening: the RACER trial study design

**DOI:** 10.1186/s13063-019-3867-5

**Published:** 2019-12-23

**Authors:** L. M. F. H. Neeter, I. P. L. Houben, P. J. Nelemans, T. J. A. Van Nijnatten, R. M. Pijnappel, C. Frotscher, M. Osinga-de Jong, F. Sanders, T. Van Dalen, H. P. J. Raat, B. A. B. Essers, J. E. Wildberger, M. L. Smidt, M. B. I. Lobbes

**Affiliations:** 1 Department of Radiology and Nuclear Medicine, Maastricht University Medical Center+, P.O. Box 5800, 6202 AZ Maastricht, the Netherlands; 2 GROW School for Oncology and Developmental Biology, Maastricht University, Maastricht, the Netherlands; 3 Department of Medical Imaging, Zuyderland Medical Center, Sittard-Geleen, the Netherlands; 4 Department of Epidemiology, Maastricht University, Maastricht, the Netherlands; 5 Department of Radiology, University Medical Center Utrecht, Utrecht, the Netherlands; 6 Department of Radiology, Diakonessenhuis, Utrecht, the Netherlands; 7 Department of Surgery, Diakonessenhuis, Utrecht, the Netherlands; 8 Department of Radiology, Laurentius Hospital, Roermond, the Netherlands; 9 Department of Clinical Epidemiology and Medical Technology Assessment, Maastricht University Medical Center+, Maastricht, the Netherlands; 10 Department of Surgery, Maastricht University Medical Center+, Maastricht, the Netherlands

**Keywords:** Breast, Mammography, Contrast-enhanced spectral mammography, Screening, Cancer

## Abstract

**Background:**

In the Dutch breast cancer screening program, women recalled with a BI-RADS 0 score are referred for additional imaging, while those with BI-RADS 4/5 scores are also directed to an outpatient breast clinic. Approximately six out of ten women are recalled without being diagnosed with a malignancy. However, these recalls require additional imaging and doctor visits, which result in patient anxiety and increased health care costs. Conventional types of imaging used for additional imaging are full-field digital mammography and tomosynthesis. Contrast-enhanced spectral mammography has proved to have higher sensitivity and specificity than conventional imaging in women recalled from screening. Therefore, the aim is to study if CESM instead of conventional imaging is a more accurate, patient-friendly, and cost-effective strategy in the work-up of women recalled from breast cancer screening.

**Methods:**

This prospective, multicenter, randomized controlled trial will be conducted at four centers and will include 528 patients recalled for suspicious breast lesions from the Dutch breast cancer screening program. Participants are randomized in two groups: (1) standard care using conventional breast imaging techniques as initial imaging after recall versus (2) work-up primarily based on CESM. Written informed consent will be collected prior to study inclusion. The primary outcome is the diagnostic accuracy for detection of breast cancer. Secondary outcomes are numbers of additional diagnostic exams, days until final diagnosis, health care costs, and experienced patient anxiety.

**Discussion:**

Based on previously published retrospective studies, we expect to demonstrate in this prospective multicenter randomized controlled trial, that using CESM as a primary work-up tool in women recalled from breast cancer screening is a more accurate, cost-effective, and patient-friendly strategy.

**Trial registration:**

Netherlands Trial Register, NL6413/NTR6589. Registered on 6 July, 2017.

## Background

In the Netherlands, a national breast cancer screening program was introduced in 1990, with full implementation being completed in 1997. All women aged 50–75 years receive an invitation to participate biennially. At mobile screening units, full-field digital mammography (FFDM) is performed. By default, two images are made per breast, one in cranio-caudal (CC) view and one in mediolateral oblique (MLO) view. The images are independently screened for suspicious lesions by two certified screening radiologists. In the case of discrepancy, a third (senior and unblinded) radiologist will make the final decision regarding referral. In the screening recalls, a distinction is made between Breast Imaging Reporting and Data System (BI-RADS) 0 score and BI-RADS 4 or 5 score recalls [1]. Women recalled with a BI-RADS 0 score are directly referred by their general physician (GP) for additional imaging. Women recalled with a BI-RADS 4 or 5 score are first referred by their GP to an outpatient breast clinic, before being directed to a radiology department for additional imaging [2].

Conventional additional imaging may consist of additional (special) FFDM images or digital breast tomosynthesis (DBT), which can be combined with other imaging modalities, such as breast ultrasound (US). Based on the findings during the imaging work-up, tissue sampling might be recommended: either core needle biopsy (CNB), vacuum-assisted biopsy (VAB), or fine-needle aspiration cytology (FNAC). The total work-up in the evaluation of the recalled women can also be extended with breast magnetic resonance imaging (MRI) (e.g., in inconclusive findings) or 6 and/or 12 months follow-up with FFDM, DBT, and/or US.

Approximately six out of ten recalled women from the Dutch breast screening program are not diagnosed with breast cancer [3]. These recalls generate patient distress and anxiety as well as additional doctor visits, medical imaging, and health care costs [3–5]. Also, participation rates for subsequent screening rounds decrease after a recall of women without malignancy [6].

Prior retrospective studies have shown that for these women contrast-enhanced spectral mammography (CESM) proved to be an adequate problem-solving tool, reducing the number of false positives while maintaining high sensitivity [3, 5]. Hypothetically, the use of CESM as an imaging work-up in women recalled from screening reduces the number of additional imaging exams and follow-up doctor visits, potentially saving health care costs [7]. Especially for women with dense breasts, CESM seems to be of additional value, since these women are more at risk of undergoing additional imaging or follow-up after recall due to the low sensitivity of FFDM [8, 9]. Moreover, Houben et al. showed that occult breast cancers are detected when using CESM in up to 4% of women in this population, increasing overall accuracy [10].

Therefore, we propose a prospective, multicenter, randomized controlled trial, aiming to study whether a work-up using CESM instead of conventional imaging modalities such as FFDM or DBT (which is the current standard of care) for women recalled from screening is a more accurate, more patient-friendly, and more cost-effective strategy.

## Methods

The Rapid Access to Contrast-Enhanced spectral mammogRaphy (RACER) study is a multicenter, prospective, randomized controlled clinical trial. Participants will be randomized in two study arms: (1) a control group undergoing standard care, i.e., work-up of recalled women based on conventional imaging (such as FFDM, US, DBT, or MRI) versus (2) work-up primarily based on CESM findings. The follow-up period is 2 years, until the next screening round. Four centers will participate in patient inclusion: the coordinating center Maastricht University Medical Center+ (Maastricht), Zuyderland Medical Center (Sittard-Geleen), Diakonessenhuis (Utrecht), and Laurentius Hospital (Roermond). The study is coordinated (study design, protocol, trial master file, case report forms, and ethical approval) by the research team in Maastricht including the Principal Investigator and Study Coordinator. Each center has a breast radiologist or surgeon as lead investigator and is assisted by other radiologists and technicians in that center for patient inclusion with randomization. Training of new research team members will be done by the Study Coordinator. The Study Coordinator also assists with and is responsible for correct data entry in all centers. Central ethical approval has been confirmed from the Medical Research Ethics Committee of University hospital Maastricht and Maastricht University (decision no. METC171082/NL62788.068.17), and we will not begin recruiting at other centers in the trial until local ethical approval has been obtained.

A data management system with electronic case report forms (eCRFs) is used to manage the clinical data of the participants in anonymous form by their trial identification number. No biological specimens will be collected in this trial. Data entry, access, and storage are restricted to the research teams, and this is monitored. Auditing, including site visits, will be performed by the Clinical Trial Center Maastricht (CTCM) at each center before, during, and at the end of the study. Since this study is marked as a “low-risk study” by the CTCM, a data monitoring committee is not commissioned. Interim analysis and premature termination of the study are not applicable; however, periodic trial progress reports are requested by the main funders of the study. After completion of the study, each center will store all their study data for 15 years.

### Study population

All women recalled for a suspicious breast lesion from our national screening program are eligible for inclusion. Inclusion criteria are the ability to provide written informed consent and being recalled from breast cancer screening during the 18-month study inclusion period. Excluded are women with a known allergy to iodine-based contrast agents and women at risk for developing contrast-induced nephropathy or women with known renal insufficiency, according to the current guidelines.

### Primary and secondary outcomes

The primary outcome will be the accuracy assessed by diagnostic performance parameters, such as sensitivity, specificity, positive predictive value (PPV), negative predictive value (NPV), receiver operating characteristics (ROC) curve, and area under the curve (AUC). Radiologists will prospectively provide a single BI-RADS classification for each exam, where a BI-RADS score of 1–3 will be considered ”benign” and 4-5 ”malignant”. The final BI-RADS score (BI-RADS 1–5), based on imaging, will be compared with the BI-RADS score given by the screening program. The accuracy assessed by diagnostic performance parameters will be assessed after the next screening round after approximately 2 years.

Secondary outcomes will be quality of life (QoL), days until final diagnosis, cost-effectiveness, and experienced patient anxiety during a follow-up of 18 months. Three validated questionnaires will be presented at six different time points (at study inclusion; after 2 and 4 weeks; and after 6, 12, and 18 months). QoL will be assessed by the Dutch version of the EuroQol five-dimension, five-level (EQ-5D-5 L) questionnaire, including a visual analog scale (EQ-VAS). This questionnaire is a preference-based instrument used to value health states [11]. The Dutch tariff for the EQ-5D-5L questionnaire, as established by Versteegh et al., will be used to calculate utility scores per health state [12]. Then the quality-adjusted life years (QALYs) will be modeled based on these utility scores. Resource use related to diagnostics will be collected during the trial.

Health-related anxiety, both state and trait anxiety, will be measured by the Dutch version of the State Trait Anxiety Inventory (STAI-DY-1 and STAI-DY-2) questionnaires. Each STAI has 20 items and will be rated on a 4-point Likert scale, scoring 20–80 points per STAI. A higher score corresponds to a higher anxiety level [13].

**Fig. 1:**
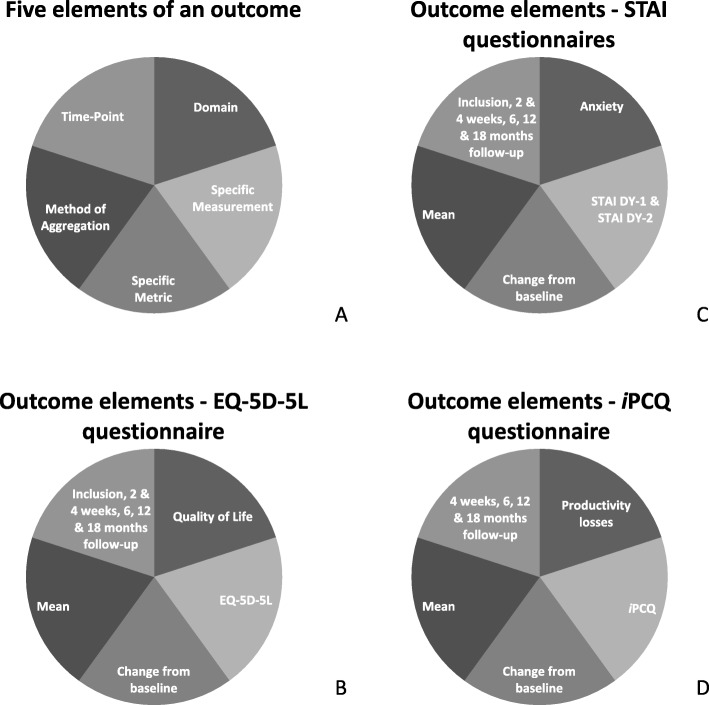
**a** Schematic overview of an outcome specified in the five elements defined by Saldanha et al. [14]. **b**–**d** Five elements of the outcome of, respectively, the EQ-5D-5 L, STAI, and iMTA Productivity Costs Questionnaire (iPCQ)

For the QoL and STAI scores, inter- and intra-patient differences over time from baseline will be compared including those between the intervention and control group. The scores will also be compared between the women with a follow-up exam after 6 or 12 months and those without this follow-up. Figure 1 shows the outcomes defined by the five outcome elements described by Saldanha et al. [14].

To reduce patient effort, the questionnaires are offered digitally, or by telephone, if desired by the participant. Data from the digital questionnaires is automatically stored in the data management system.

### Informed consent and randomization protocol

All recalled women announced at one of the participating centers will be contacted for study participation. Written informed consent will be obtained and the first EQ-5D-5 L and STAI questionnaires regarding anxiety and current health state will be completed before any other study procedure is carried out. Participants are also asked for their consent to use their data in possible future studies. See Additional file 1 for the informed consent form in Dutch.

Women will be randomized to undergo standard of care (control arm) or CESM (intervention arm). Minimization with stratification factors will be applied in the computer-generated randomization screening and enrollment application software ALEA (version 3.0.2083.212r; ALEA Clinical, Abcoude, the Netherlands). Randomization will be stratified by the following: predominant reason for recall (mass, calcifications, asymmetry, architectural distortion), recall BI-RADS score (BI-RADS 0 versus BI-RADS 4/5), and study center. Enrollment in the four centers, including the master randomization file, will be overseen by the Study Coordinator. During the inclusion visit, written consent will be conducted and data entry will be done by one of the research team members in each center to execute randomization using ALEA. Should a problem occur in including a patient or randomization, the Study Coordinator must be reached so that he can provide assistance. After allocation, patient and radiologist will be informed about the outcome; hence, they are not blinded. However, the radiologist is blinded for the outcomes between the two groups, since the final diagnosis and pathology are not yet known. The outcome assessors and data analysts are blinded for the judgment of the next screening round by the screening radiologists, since accuracy also depends on the next screening round outcome. Each participant is assigned a trial identification number for anonymization, to analyze the data, and further to be used in publications. In each participant’s hospital patient file a statement will be noted about study participation and the assigned intervention or control group. On request, study participation can be ended during the follow-up phase.

### The control group: usual care with conventional breast imaging

Women undergoing standard of care will have their screening FFDM re-evaluated, adding (special) FFDM views, DBT, or US if deemed necessary. In FFDM and DBT, the breast is compressed between a paddle and detector plate. The laterality of the breast and views per breast depend on the judgment of the radiologist. At least one additional view of the recalled side will be performed. Tissue sampling (CNB, VAB, FNAC) can be recommended to support or to invalidate suspicious findings on imaging. In case of inconclusive findings, the radiologist can consider follow-up in 6 and/or 12 months or single breast MRI. The use of CESM is not permitted in this study arm.

### The experimental group: contrast-enhanced spectral mammography

CESM is based on visualizing angiogenesis in tumor tissue using dual-energy mammography [15]. Prior to image acquisition, an intravenous catheter will be placed in the antecubital vein, after which its patency will be checked by a saline test bolus. A non-ionic, low-osmolar contrast agent consisting of either iopromide (Ultravist® 300, Bayer Healthcare, Berlin, Germany) or iobitridol (Xenetix® 300, Guerbet, Villepinte, France) will be administered at a dose of 1.5 mL/kg body weight. If an automatic injector is used instead of manual administration, the injection rate will be 2.5–3 mL/s, followed by a saline flush. Image acquisition is started after at least 2 min after contrast administration [16]. Although there is a limited risk of complications, such as hematoma or incorrect catheter placement, a previous retrospective study showed that the risks of adverse reactions to the contrast agents and contrast-induced nephropathy are negligible [17]. Two days after CESM, the patient file will be consulted to investigate whether any late (serious) adverse reactions to the contrast agent have occurred that need medical treatment.

**Fig. 2:**
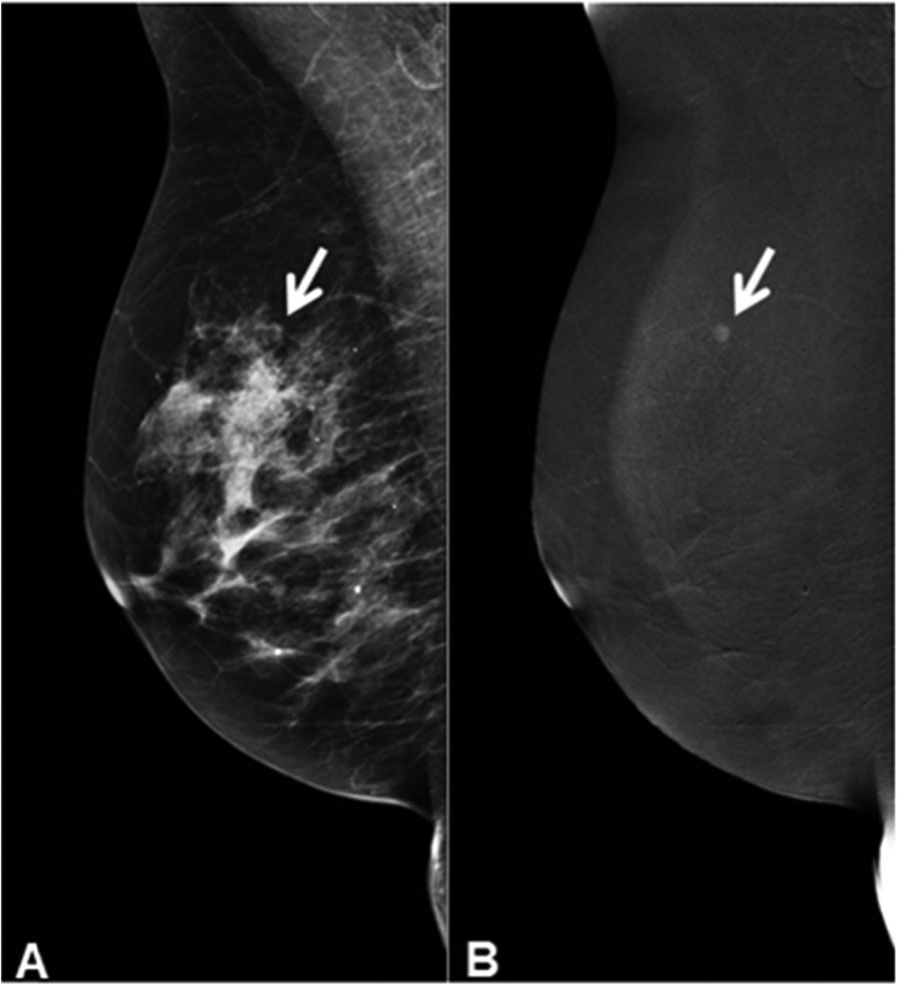
CESM image of right breast in MLO view. **a** Low-energy image, which is equivalent to FFDM image. **b** Recombined image. The white arrows indicate the suspect mass, which is only visible on the recombined image and not in the low-energy image. The high-energy image is not shown since it is not of clinical value. Histopathological findings showed grade I invasive carcinoma

Similar to FFDM, the breasts are positioned between a mammography paddle and detector plate, and the four standard views are made: a cranio-caudal (CC) and mediolateral oblique (MLO) view of each breast. Special views can be requested by the reviewing radiologist. A typical CESM exam consists (per breast exposure) of two images: a low- and a high-energy image, which are acquired within seconds [18]. These images are used in post-processing to acquire the recombined image, which shows areas of contrast accumulation (Fig. 2). The CESM image acquisition needs to be completed within 10–12 min after contrast administration [19]. As in the control group, US, tissue sampling, MRI, and follow-up can be considered in case of inconclusive findings.

### Establishment of final diagnosis (the reference standard)

For the control group, final diagnoses of the (recalled) breast lesions can be divided into four categories [3, 5]: (1) simple cysts; (2) superposition densities; (3) solid, benign masses; (4) (invasive) breast cancer or ductal carcinoma in situ (DCIS). The first three groups are defined as benign findings. To ascertain a final diagnosis of (1), targeted US is performed, followed by cyst aspiration and a second FFDM of that breast to confirm that the lesion has disappeared. Final diagnosis of (2) requires a minimum of one special view or DBT, including targeted US. To rule out false negative (FN) findings, 6 and/or 12 months follow-up or breast MRI can be considered. Final diagnosis for categories (3) and (4) is acquired with US-guided or stereotactic tissue sampling. If cancer or DCIS is diagnosed, the subject will have a ”true positive” (TP) finding. In subjects where no breast cancer or DCIS is diagnosed, the follow-up period of 2 years will determine the true disease status. If no interval cancers have been detected and the subject has not been recalled in the subsequent screening round, the case will be considered a ”true negative” (TN).

In the CESM group, diagnoses are acquired slightly differently. For (1), CESM will show an ”eclipse sign” which is pathognomonic for cysts [3]. No further action is needed. For category (2), a negative CESM exam with no suspect lesion on both low-energy and recombined images rules out (pre)malignant lesions due to CESM’s high negative predictive value [3, 5]. To acquire final diagnoses of (3) and (4), CESM and targeted US are performed including tissue sampling for pathological confirmation.

Since most benign recalls are caused by cysts or superposition densities, the investigated intervention will most likely result in fewer additional exams and tissue samplings among these recalls [3, 5]. Moreover, follow-up exams can be omitted, which is more patient-friendly, and hypothetically, more women will attend the subsequent screening round when they are examined with CESM.

**Fig. 3:**
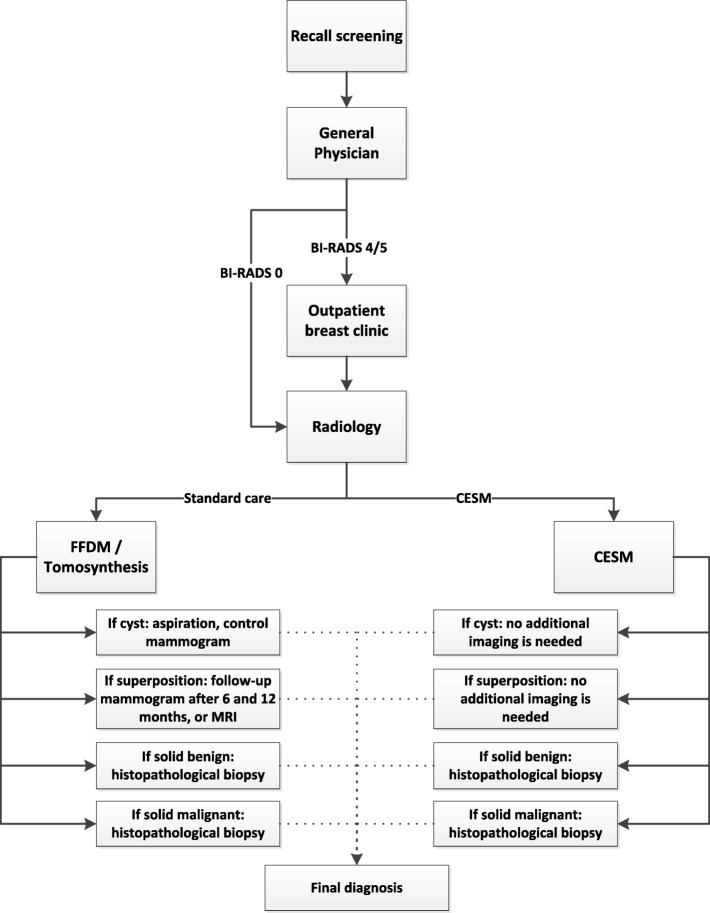
Flow chart of work-up RACER study. *Left arm*: standard care group; *right arm*: CESM group

A flow chart is presented in Fig. 3, summarizing the study design, the randomization process, and the establishment of the different diagnoses.

### Sample size calculation

Prior research showed a specificity of FFDM of 40% in this population [5]. To enable detection of a clinically relevant increase of specificity by 15% (from 40% to 55%), 176 patients without malignant disease per group are required (power 80%, alpha = 5%). The prevalence of malignant lesions in women recalled from the breast screening program is about 30%; thus, 70% have benign lesions. Therefore, a total of 251 women per group (176/0.7) has to be included. To account for 5% loss to follow-up, the final number of patients to be included is 528 (502/0.95). The calculations were done with OpenEpi [20].

### Statistical analysis

The primary objective of the study is to demonstrate that the use of CESM as a work-up tool for women recalled from breast cancer screening is more accurate compared to standard care consisting of conventional imaging. The secondary objectives are to evaluate whether this novel approach is a more patient-friendly and cost-effective strategy in the work-up of women recalled from breast cancer screening, requiring fewer days until final diagnosis and less additional imaging.

Radiologists will prospectively provide a single BI-RADS classification for each exam, where a BI-RADS score of 1–3 will be considered ”benign” and 4-5 ”malignant”. Based on this cutoff, the final BI-RADS score and recall BI-RADS score, the diagnostic performance parameters sensitivity, specificity, PPV, NPV, and AUC under the ROC curve will be assessed as the primary endpoint for both study arms. Differences in proportions between the two randomized groups will be tested for significance with a chi-square test, and the difference in AUCs will be tested using the method proposed by Hanley et al. [21].

Secondary outcomes are QoL, patient anxiety, and cost-effectiveness. The course of scores on QoL with QALY and patient anxiety (STAI) from baseline over time at the six time points will be visualized and differences between groups will be tested using mixed linear models, which account for correlations between repeated measurements. Single and multiple imputation will be used to replace missing data.

Both intention-to-treat and per protocol analyses will be performed. The per protocol population for the primary outcomes are those who got imaging exams based on their group allocation. Women who underwent CESM in the standard care group are excluded from the per protocol analysis. For the secondary outcomes this population can be further specified to those with questionnaires completed at six time points. *P* values < 0.05 will be considered to indicate statistical significance. Analyses will be performed with SPSS (version 25, IBM Corporation, Armonk, NY, USA) and STATA (version 15, StataCorp LLC, College Station, TX, USA).

### Economic evaluation

Decision-analytic modeling will be applied to estimate the cost-effectiveness of CESM compared to conventional imaging modalities FFDM or DBT for recalled women from the Dutch national screening program. The economic evaluation will be performed from both a health care and a societal perspective. Cost-effectiveness will be expressed as the incremental costs per QALY gained as outcome measure [22]. All resource use related to diagnostics will be registered. In addition, productivity loss will be measured with the iMTA Productivity Costs Questionnaire (iPCQ) at 4 weeks and at 6, 12, and 18 months [23]. Reference prices will be obtained from the Dutch manual for costing research, hospital financial department, or the literature [24]. Uncertainty surrounding the incremental costs per QALY will be analyzed with a non-parametric bootstrap analysis [25]. Results of the bootstrap analysis will be presented in cost-effectiveness planes and acceptability curves. A probabilistic sensitivity analysis will be performed to examine different parameter uncertainties.

**Fig. 4:**
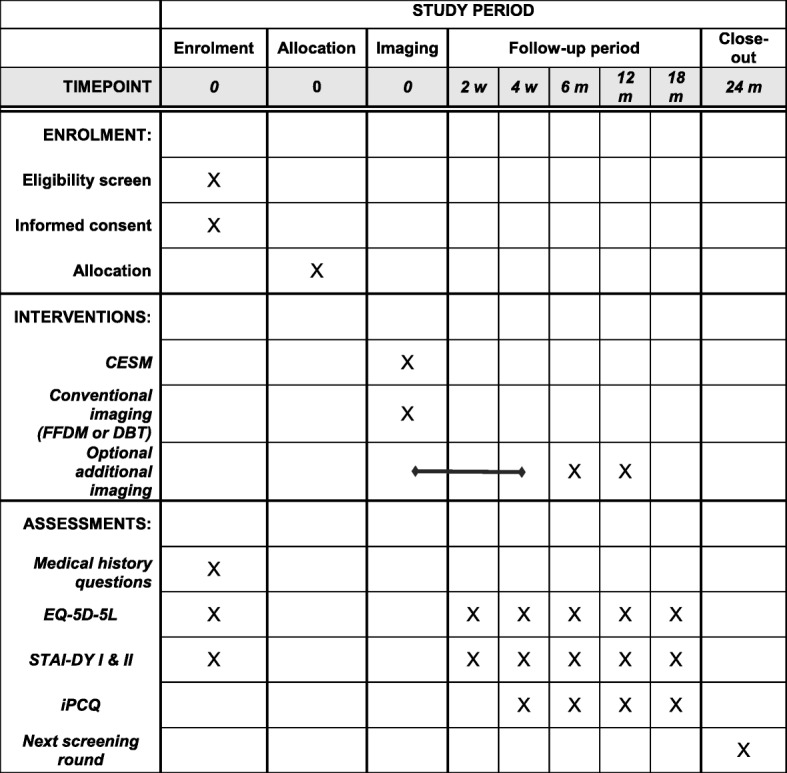
Schedule of study activities according to Standard Protocol Items: Recommendations for Interventional Trials (SPIRIT)

In Fig. 4 an overview, according to Standard Protocol Items: Recommendations for Interventional Trials (SPIRIT), is given of all study activities, including the iPCQ assessments.

## Discussion

We present the rationale and design of the RACER study, which is a multicenter, prospective, randomized controlled trial investigating the work-up after breast cancer screening referral. This design is in accordance with the SPIRIT guidelines (Additional file 2).

Recent studies have consistently shown that CESM is superior to FFDM, even when the latter is combined with targeted US [26–29]. Lobbes et al. showed that in women recalled from screening, the detection of breast cancer was comparable between the two techniques, but specificity almost doubled from 42.0 to 87.7% when using CESM as a work-up tool [3]. In other words, fewer false positive findings were observed, and with the high negative predictive value of 97.1% in this study, there was a high ability to rule out breast cancer if the CESM showed a negative exam. These initial findings were later confirmed by Lalji et al. in 199 new cases, analyzed by a panel of ten different radiologists [5]. In this study, radiologists without any CESM experience (but with extensive experience in reading FFDM exams) performed as well as radiologists with CESM experience. Reading a CESM exam does not require a dedicated learning curve. Radiologists in the participating centers will have different levels of experience in reading CESM exams. However, these works used a retrospective study design. Also, many other studies on CESM are retrospective in nature. Only some studies collect data prospectively, and most have not done so in a randomized controlled trial like the RACER study.

The RACER study has several strengths. First, a direct comparison between CESM and FFDM, both with targeted US allowed, is possible. Until now, some studies have investigated the comparison between CESM versus FFDM plus targeted US [27, 28]. However, in clinical practice, suspicious findings on CESM are further evaluated with US as well. Klang et al. recently showed that in BI-RADS 3 lesions detected on CESM, targeted US could be used to determine the necessity to perform tissue sampling [30]. Although CESM is superior to FFDM plus US, the difference in diagnostic accuracy is expected to increase again when CESM/US is compared with FFDM/US. To the best of our knowledge, this has not been studied before in a prospective study design.

Second, the patients’ mental state during the recall and diagnostic work-up is thoroughly assessed by validated questionnaires. Due to the higher diagnostic performance of CESM, especially in terms of reducing the number of false positive findings, it is expected that in the intervention arm fewer additional exams will be performed, such as follow-up after 6 or 12 months, or breast MRI. These additional exams cause a delay in the assessment of final diagnosis and increase health care costs; plus we hypothesize that this will influence a patient’s mental state during these months. Finally, the study design allows for a cost-effectiveness analysis, showing us the impact on health care costs of both strategies during this period. We hypothesize that using CESM as a work-up tool is more efficient and cost-effective than following current standard care. If proven to be cost-effective, an important hurdle is taken toward acquiring reimbursement by health insurance companies for CESM, which at present frustrates the further introduction of CESM worldwide.

Using CESM as a work-up tool also has some limitations. The most important disadvantages are the increased radiation dose and the administration of iodine-based contrast agents. Regarding the increase in radiation dose, several studies have shown that the mean radiation dose of CESM per exposure is in the range of 2.5-2.8 mGy [18, 31–33]. In comparison, the radiation dose of FFDM in these studies varied from 1.4 to 1.8 mGy. Jeukens et al. showed that the CESM radiation dose is approximately 80% higher than that in FFDM: 2.8 mGy versus 1.6 mGy, respectively [18]. Nevertheless, it is still within internationally accepted limits and does not substantially increase breast cancer incidence or mortality in women recalled from screening, who are at least 50 years. We believe that this increased dose is justified in the dedicated population as outlined. The chance of acquiring breast cancer due to this radiation exposure is expressed in the life attributable risk (LAR) numbers. For example, for a single view CESM acquisition having a mean radiation dose of 2.8 mGy per exposure, the LAR number for cancer incidence is 0.4 in 100,000 persons at the age of 60 years [34]. The LAR number for cancer mortality at this age is even 2–3 times lower. Some even advocate that there is no effect of radiation under a dose of 100 mSv. Hence, one may conclude that CESM exposure poses only a small additional risk compared to the lifetime risk for breast cancer incidence and mortality of 12,000 and 3000 cases per 100,000 women, respectively.

Regarding the use of iodine-based contrast, Houben et al. had in clinical practice in a similar population a low incidence (0.6%) of adverse hypersensitivity reactions [10]. In contrast, they also showed that occult cancers are being detected in 3–4% of the recalled women by using CESM. Consequently, the chance of finding occult (small) breast cancers is higher than the chance of having adverse reactions to contrast administrations (which are all documented in the RACER study), which in our opinion would justify the use of a CESM-based work-up of women recalled from screening.

In short, we believe that the prospective multicenter randomized controlled RACER study will show that in recalled women a work-up based on CESM will be more accurate than usual care (based on conventional imaging such as FFDM). Higher specificity at similar sensitivity will reduce false positives and the volumes of additional diagnostic exams required to reach a final diagnosis, ultimately leading to a decrease in health care costs and patient-experienced anxiety during this period.

### Trial status

Participants are currently enrolled via protocol version number 6.1 (April 23rd 2019). The study protocol was approved by the Medical Ethics Review Committee of Maastricht University Medical Center+ in January 2018. The first participants were randomized in April 2018. Enrollment is expected to be completed before January 1st, 2021.

## Supplementary information

**Additional file 1.** Informed consent form RACER study in Dutch.

**Additional file 2.** Standard Protocol Items: Recommendations for Interventional Trials (SPIRIT) 2013 checklist.

## Data Availability

The database, once the project has ended, will be shared on request and after approval by the study committee. A part of the collected imaging and clinical data, for which the patients will explicitly give informed consent, will be shared with The D-Lab: Decision Support for Precision Medicine (GROW School for Oncology and Developmental Biology, Maastricht University Medical Center) to be used in an additional artificial Intelligence analysis.

## Abbreviations

AUC: Area under the curve
BI-RADS: Breast Imaging Reporting and Data System
CC: Cranio-caudal
CESM: Contrast-enhanced spectral mammography
CNB: Core needle biopsy
CTCM: Clinical Trial Center Maastricht
DBT: Digital breast tomosynthesis
DCIS: Ductal carcinoma in situ
eCRF: Electronic case report form
FFDM: Full-field digital mammography
FN: False negative (findings)
FNAC: Fine-needle aspiration cytology
GP: General physician
*i*PCQ: iMTA Productivity Costs Questionnaire
LAR: Life attributable risk
MLO: Mediolateral oblique
MRI: Magnetic resonance imaging
NPV: Negative predictive value
PPV: Positive predictive value
QALY: Quality-adjusted life year
QoL: Quality of life
ROC: Receiver operating characteristics (curve)
STAI: State Trait Anxiety Inventory
TN: True negative findings
TP: True positive
US: Ultrasound
VAB: Vacuum-assisted biopsy
