# Supplementary material for: Rapid Access to Contrast-Enhanced spectral mammogRaphy in women recalled from breast cancer screening: the RACER trial study design
Source: Trials. 2019 Dec 23;20:759. doi: 10.1186/s13063-019-3867-5 (PMC6929439; doi:10.1186/s13063-019-3867-5)
Supplement: Supplementary file 1 — Additional file 1. Informed consent form RACER study in Dutch. [file 13063_2019_3867_MOESM1_ESM.docx]

**Toestemmingsformulier**

**‘Snelle toegang tot Contrast-Enhanced Spectral Mammography (RACER studie): een efficiëntere work-up van vrouwen verwezen door het Bevolkingsonderzoek naar borstkanker’**

*‘Rapid Access to Contrast-Enhanced spectral mammogRaphy (RACER): a more efficient work-up of women recalled from breast cancer screening’*

Ik bevestig, dat ik het informatieformulier voor de proefpersoon heb gelezen. Ik begrijp de informatie. Ik heb de gelegenheid gehad om aanvullende vragen te stellen. Deze vragen zijn naar tevredenheid beantwoord. Ik heb voldoende tijd gehad om over deelname na te denken.

Ik weet dat mijn deelname geheel vrijwillig is en dat ik mijn toestemming op ieder moment kan intrekken zonder dat ik daarvoor een reden hoef te geven.

Ik geef toestemming om mijn huisarts op de hoogte te brengen van mijn deelname aan dit onderzoek.

Ik geef toestemming dat leden van het onderzoeksteam inzage kunnen krijgen in mijn medische gegevens en onderzoeksgegevens.

Ik geef toestemming de behandelend specialist op de hoogte te brengen van deelname aan dit onderzoek.

Ik geef toestemming voor het verzamelen en gebruiken van mijn gegevens voor de beantwoording van de onderzoeksvragen in dit onderzoek.

Ik geef wel/geen* toestemming om de gegevens te gebruiken voor de extra beeldanalyse.

Ik geef wel/geen* toestemming om de gegevens te gebruiken voor toekomstige onderzoeken (anders dan de extra beeldanalyse).

Ik weet dat voor de controle van het onderzoek sommige mensen toegang tot al mijn gegevens kunnen krijgen. Die mensen staan vermeld in deze informatiebrief. Ik geef toestemming voor die inzage door deze personen.

Ik geef toestemming om mijn gegevens gedurende maximaal 15 jaar na afloop van de studie te bewaren.

Ik wil meedoen aan dit onderzoek

Naam patiënt :___________________________________________________________

Handtekening :_______________________________________Datum : ___ / ___ / __

Ik verklaar hierbij dat ik bovengenoemde persoon volledig heb geïnformeerd over het genoemde onderzoek. Als er tijdens het onderzoek informatie bekend wordt die deze toestemming zou kunnen beïnvloeden, breng ik de proefpersoon hiervan tijdig op de hoogte.

Naam onderzoeker :_______________________________________________________

Handtekening :_______________________________________Datum : ___ / ___ / __

**Doorhalen wat niet van toepassing is.*

**Exemplaar voor de patiënt**

*Toestemmingsverklaring versie 5.0, 1 maart 2019; ToetsingOnline nummer NL62788.068.17*

**Toestemmingsformulier**

**‘Snelle toegang tot Contrast-Enhanced Spectral Mammography (RACER studie): een efficiëntere work-up van vrouwen verwezen door het Bevolkingsonderzoek naar borstkanker’**

*‘Rapid Access to Contrast-Enhanced spectral mammogRaphy (RACER): a more efficient work-up of women recalled from breast cancer screening’*

Ik bevestig, dat ik het informatieformulier voor de proefpersoon heb gelezen. Ik begrijp de informatie. Ik heb de gelegenheid gehad om aanvullende vragen te stellen. Deze vragen zijn naar tevredenheid beantwoord. Ik heb voldoende tijd gehad om over deelname na te denken.

Ik weet dat mijn deelname geheel vrijwillig is en dat ik mijn toestemming op ieder moment kan intrekken zonder dat ik daarvoor een reden hoef te geven.

Ik geef toestemming om mijn huisarts op de hoogte te brengen van mijn deelname aan dit onderzoek.

Ik geef toestemming dat leden van het onderzoeksteam inzage kunnen krijgen in mijn medische gegevens en onderzoeksgegevens.

Ik geef toestemming de behandelend specialist op de hoogte te brengen van deelname aan dit onderzoek.

Ik geef toestemming voor het verzamelen en gebruiken van mijn gegevens voor de beantwoording van de onderzoeksvragen in dit onderzoek.

Ik geef wel/geen* toestemming om de gegevens te gebruiken voor de extra beeldanalyse.

Ik geef wel/geen* toestemming om de gegevens te gebruiken voor toekomstige onderzoeken (anders dan de extra beeldanalyse).

Ik weet dat voor de controle van het onderzoek sommige mensen toegang tot al mijn gegevens kunnen krijgen. Die mensen staan vermeld in deze informatiebrief. Ik geef toestemming voor die inzage door deze personen.

Ik geef toestemming om mijn gegevens gedurende maximaal 15 jaar na afloop van de studie te bewaren.

Ik wil meedoen aan dit onderzoek

Naam patiënt :___________________________________________________________

Handtekening :_______________________________________Datum : ___ / ___ / __

Ik verklaar hierbij dat ik bovengenoemde persoon volledig heb geïnformeerd over het genoemde onderzoek. Als er tijdens het onderzoek informatie bekend wordt die deze toestemming zou kunnen beïnvloeden, breng ik de proefpersoon hiervan tijdig op de hoogte.

Naam onderzoeker :_______________________________________________________

Handtekening :_______________________________________Datum : ___ / ___ / __

**Doorhalen wat niet van toepassing is.*

**Exemplaar voor de onderzoeker**

*Toestemmingsverklaring versie 5.0, 1 maart 2019; ToetsingOnline nummer NL62788.068.170*
